# Case Report of Gastrointestinal Bleeding in an Adult with Chronic Visceral Acid Sphingomyelinase Deficiency

**DOI:** 10.1155/2019/9613457

**Published:** 2019-04-04

**Authors:** David Cassiman, Louis Libbrecht, Wouter Meersseman, Alexander Wilmer

**Affiliations:** ^1^Department of Gastroenterology-Hepatology and Metabolic Center, University of Leuven, Belgium; ^2^Laboratory of Hepatology, University of Leuven, Belgium; ^3^Department of Pathology, University Hospital Saint-Luc, Brussels, Belgium; ^4^General Internal Medicine-Metabolic Diseases, University Hospital Gasthuisberg, Leuven, Belgium; ^5^Medical Intensive Care, University Hospital Gasthuisberg, Leuven, Belgium

## Abstract

**Introduction:**

Acid sphingomyelinase deficiency (ASMD, also known as Niemann-Pick Type A and Type B disease) is a rare, inherited metabolic disorder. Liver-related issues, including cirrhosis and variceal haemorrhage, are a leading cause of early mortality in individuals with chronic forms of ASMD. Due to the rarity of this lysosomal storage disorder, there can be a lack of awareness that adults with chronic ASMD disease are at significant risk of cirrhosis, portal hypertension, and variceal bleeding. This case highlights an unusual presentation of recurrent variceal bleeding in an adult with cirrhosis and portal hypertension due to chronic visceral ASMD.

**Case Presentation:**

A patient with severe splenomegaly was diagnosed with ASMD at age of 25. At age 64 they had multiple hospital admissions for hematochezia (originally diagnosed as ischemic colitis) accompanied by hypotension (blood pressure 91/45 mmHg), anemia (hemoglobin 8.5g/dL, ref 12-16; INR 1.4, ref ≤1.2), and mild renal insufficiency (creatinine 1.33mg/dL, ref 0.51-0.95). Colonoscopy did not reveal a source of bleeding. Computerized tomography scanning imaging showed diffuse venous collaterals and ascites. Arteriographies during subsequent episodes of bleeding were negative for active arterial intestinal bleeding. Recurrent gastrointestinal bleeding was found to originate from a varicose vein cluster connected to the right iliac vein and the superior mesenteric vein, located in the submucosa of a small intestinal loop. Multiple varices were secondary to portal hypertension in the context of cirrhosis. The patient died from recurrent variceal bleeding that exacerbated liver failure worsened by pneumonia and hypovolemic and septic shock.

**Conclusions:**

The variceal bleeding in this patient was atypical in that it originated from venous collaterals bleeding into the small intestine rather than the more typical gastroesophageal varices observed in ASMD. With long standing liver dysfunction and gradual development of portal hypertension, intestinal varices rather than occult intestinal bleeding due to ischemia should be considered in ASMD patients presenting with either hematochezia or hematemesis.

## 1. Background

Acid sphingomyelinase deficiency (ASMD, also known as Niemann-Pick Type A and Type B disease) is a rare, inherited metabolic disorder resulting in sphingolipid accumulation in nervous and visceral tissues and progressive multisystem disease [1]. Biallelic mutations in* SMPD1*, the gene encoding acid sphingomyelinase (ASM; EC3.1.4.12), give rise to a spectrum of disease phenotypes characterized by differences in the rate of disease progression and the extent of neurological and other organ system involvements. Infantile-onset neurovisceral ASMD is uniformly fatal (OMIM#25707), chronic visceral ASMD (OMIM#607616) has early childhood-adulthood onset with progressive visceral disease without neurodegeneration, and an intermediate phenotype has visceral disease and slowly progressing neurological symptoms [2]. Nonneurological manifestations include hepatosplenomegaly, liver dysfunction, dyslipidemia, cardiac valve insufficiency, interstitial lung disease, osteopenia/osteoporosis, and hematologic abnormalities [2]. There are no approved treatments for ASMD, and chronic ASMD management aims to prevent long-term complications related to liver and lung disease. Enzyme replacement therapy for chronic forms of ASMD is in clinical development [3].

Liver-related issues including cirrhosis and variceal haemorrhage are a leading cause of early mortality in individuals with chronic forms of ASMD [4–6]. Variable degrees of liver fibrosis were found in 17 adults undergoing liver biopsy during screening for enrolment in a phase 1 ASMD clinical trial, with cirrhosis present in two individuals that had no clinical symptoms of liver failure [7]. Successful liver transplantation has been performed in adults with chronic ASMD that had cirrhosis or liver failure [6, 8–10]. Since the liver is so prominently affected in ASMD, hepatologists or gastroenterologists are typically consulted and involved in management of patients with chronic ASMD forms. The following case report describes an adult with chronic visceral ASMD and an unusual presentation of gastroenterology symptoms resulting from liver disease.

## 2. Case Presentation

**Figure 1:**
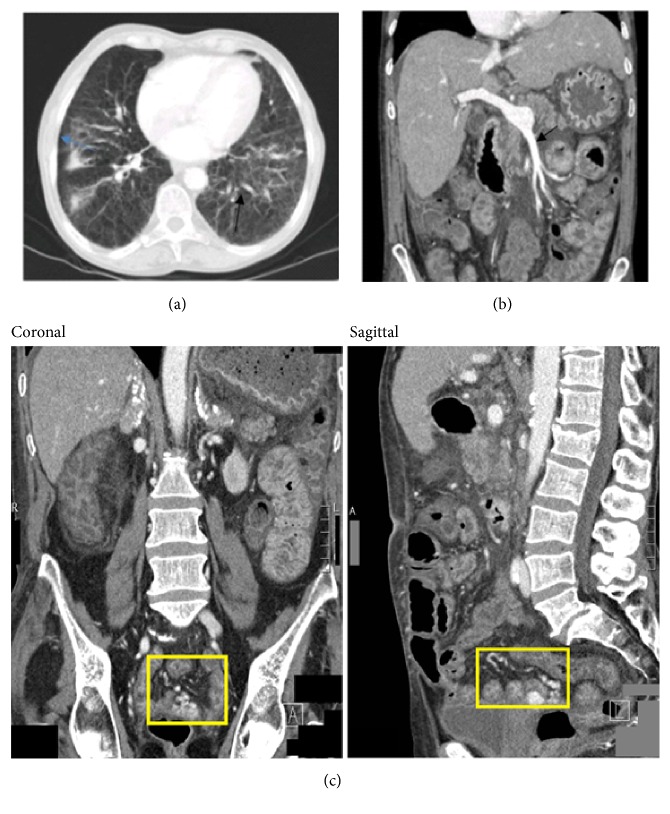
*CT of chest (a), abdomen (b), and abdomen/pelvis (c)*. (a) Chest CT showing centrilobular emphysema and signs of interstitial lung disease. There are bilateral basal inter- and intralobular septal enhancement (blue arrow) consistent with sphingomyelin deposition seen in ASMD patients. Multiple bronchiectasis (black arrow) is also present. (b) Venous phase of an abdominal contrast CT, showing an enlarged portal vein (arrow) and a moderate amount of ascites fluid in the abdominal cavity. The liver is nodular, enlarged, and steatotic. The patient is asplenic. (c) Coronal and sagittal CT images of abdomen and pelvis show the cluster of varicose veins that are contrast-enhanced (outlined by yellow boxes), situated just above the vaginal lumen, and show extravasation of contrast into the small intestinal lumen.

An adult with severe splenomegaly was diagnosed with ASMD at age of 25 while being hospitalized to undergo splenectomy for what was thought to be autoimmune thrombocytopenia. Other ASMD symptoms included hepatomegaly, thrombocytopenia, mixed dyslipidemia, interstitial lung disease (Figure 1(a)), and osteoporosis. The individual was admitted to hospital at age of 53 for* Streptococcus pneumoniae* septic shock with multiorgan failure, hepatic encephalopathy, and disseminated intravascular coagulation and received ventilation for acute respiratory distress syndrome and temporary hemodialysis for acute renal insufficiency.

At age of 64 they were admitted to hospital for hematochezia with a tentative diagnosis of ischemic colitis and admitted again one year later for hematochezia accompanied by hypotension (blood pressure 91/45 mmHg), anemia (hemoglobin 8.5g/dL, ref 12-16; INR 1.4, ref ≤1.2), and mild renal insufficiency (creatinine 1.33mg/dL, ref 0.51-0.95). Gastroscopy revealed one esophageal varix with a red spot in the absence of acute bleeding stigmata. Colonoscopy did not reveal a source of bleeding. Computerized tomography scanning imaging showed diffuse venous collaterals and ascites (Figure 1(b)). Serum-ascites albumin gradient was high (>1.1g/dL). Arteriographies during subsequent episodes of bleeding were negative for active arterial intestinal bleeding. Recurrent gastrointestinal bleeding was found to originate from a varicose vein cluster (Figure 1(c)) connected to the right iliac vein and the superior mesenteric vein, located in the submucosa of a small intestinal loop. Despite laparoscopic suturing of venous collaterals, another episode of bleeding occurred 1 month later leading to admission to the intensive care unit with hypovolemic shock, overwhelming pneumonia, deteriorating liver function/hepatic encephalopathy, anemia (hemoglobin 4.8g/dL; INR 3), lactic acidosis (lactate 9.6mmol/L, ref 0.5-2.2), and high bilirubin (7.54mg/dl, ref≤1).

**Figure 2:**
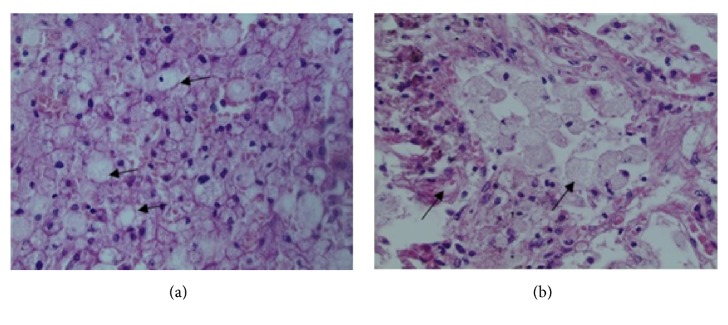
*Liver (a) and lung (b) biopsies showing presence of sphingolipid storage due to ASMD.* (a) Hepatocytes filled with vacuoles having transparent cytoplasm (arrows, hematoxylin and eosin staining, 40X). (b) Lung section showing diffuse infiltration of foamy histiocytes in alveolar spaces, as well as the interstitium, without inflammatory infiltrate (arrows, hematoxylin and eosin staining, 40X).

Cirrhosis was suspected from the clinical course and was supported by direct visualization of the liver during laparoscopy. Liver biopsy indeed showed micronodular cirrhosis with elements of nonalcoholic steatohepatitis and many hepatocytes were filled with vacuoles having transparent cytoplasm (Figure 2(a)). Hepatic venous pressure gradient was 15mmHg, consistent with portal hypertension. Deterioration of hemodynamics and liver function resulted in death. On autopsy, lung pathology showed diffuse infiltration of foamy histiocytes in alveolar spaces and interstitium without inflammatory infiltrate (Figure 2(b)).

## 3. Discussion

This case highlights an unusual presentation of recurrent variceal bleeding in an adult with cirrhosis and portal hypertension due to chronic visceral ASMD. Individuals with chronic ASMD are at risk of liver-related morbidity and mortality as identified in natural history studies [6], patients undergoing liver biopsy during screening for enrolment in clinical trials [7], and in case reports [5, 10–14]. In a study of 103 adults with chronic visceral ASMD, 9 (9%) had cirrhosis or liver failure requiring liver transplantation [6]. Liver-related issues, including cirrhosis and variceal haemorrhage, are leading causes of early mortality in individuals with chronic forms of ASMD [4].

The patient in the present case died from recurrent variceal bleeding that exacerbated liver failure worsened by pneumonia and hypovolemic and septic shock. Multiple varices were secondary to portal hypertension in the context of cirrhosis. The variceal bleeding was atypical in that it originated from venous collaterals bleeding into the small intestine rather than the more typical hematemesis observed in ASMD. With long standing liver dysfunction and gradual development of portal hypertension, intestinal varices rather than occult intestinal bleeding due to ischemia should be considered in ASMD patients presenting with either hematochezia or hematemesis. Individuals with chronic ASMD, especially asplenic patients, should be monitored for varices at all areas of risk including the most common locations such as the gastroesophageal junction and rectum, as well as less common locations including paraumbilical, splenorenal, gastrorenal, gall bladder, and, as in this case, intestinal. Identification of venous collaterals by ultrasound or CT/MRI imaging, screening for esophageal varices according to standard practices for patients with cirrhosis [15] (e.g., endoscopic screening for varices at the time of diagnosis with follow-up every 2-3 years if negative), and nonselective beta blocker therapy to lower portal pressure are recommended. Clinical monitoring and hepatic assessments for chronic visceral ASMD are described in Wasserstein et al. [16].

Due to low awareness of ASMD, the initial surgical liver biopsy assessment of vacuoles for this patient was nonalcoholic steatohepatitis, since vacuoles were interpreted as (microvesicular) steatosis, rather than sphingolipid storage due to ASMD. Similar misclassification has occurred in cholesterol ester storage disease, where lack of awareness among pathologists has resulted in misdiagnoses of nonalcoholic fatty liver disease/steatohepatitis or cryptogenic cirrhosis [17].

## 4. Conclusion

Due to the rarity of this lysosomal storage disorder, there can be a lack of awareness among gastroenterologists and hepatologists that adults with chronic ASMD disease are at significant risk of cirrhosis, portal hypertension, and variceal bleeding. This case highlights the fact that adults with ASMD and severe liver dysfunction may develop variceal bleeding at multiple sites in the intestine and viscera rather than more commonly occurring gastroesophageal varices. With long standing liver dysfunction and gradual development of portal hypertension, intestinal varices rather than occult intestinal bleeding due to ischemia should be considered in ASMD patients presenting with either hematochezia or hematemesis.

## Abbreviations

ASM: Acid sphingomyelinase
ASMD: Acid sphingomyelinase deficiency
INR: International normalized ratio
*SMPD1*: Sphingomyelin phosphodiesterase 1 gene.
